# *Psoralea corylifolia* L. Seed Extract Ameliorates Streptozotocin-Induced Diabetes in Mice by Inhibition of Oxidative Stress

**DOI:** 10.1155/2014/897296

**Published:** 2014-04-03

**Authors:** Eunhui Seo, Eun-Kyu Lee, Cheol Soon Lee, Kwang-Hoon Chun, Mi-Young Lee, Hee-Sook Jun

**Affiliations:** ^1^College of Pharmacy and Gachon Institute of Pharmaceutical Science, Gachon University, Incheon 406-840, Republic of Korea; ^2^Lee Gil Ya Cancer and Diabetes Institute, Gachon University, Incheon 406-840, Republic of Korea; ^3^Nanomol Inc. and Regen Phoenix R&D Institute, Incheon 406-840, Republic of Korea; ^4^KM-Based Herbal Drug Research Group, Korea Institute of Oriental Medicine, Daejeon 305-811, Republic of Korea; ^5^Gachon Medical Research Institute, Gil Hospital, Incheon 405-760, Republic of Korea

## Abstract

Pancreatic beta-cell death is known to be the cause of deficient insulin production in diabetes mellitus. Oxidative stress is one of the major causes of beta-cell death. In this study, we investigated the effects of *Psoralea corylifolia* L. seed (PCS) extract on beta-cell death. Oral administration of PCS extract resulted in a significant improvement of hyperglycemia in streptozotocin-induced diabetic mice. PCS extract treatment improved glucose tolerance and increased serum insulin levels. To study the mechanisms involved, we investigated the effects of PCS extract on H_2_O_2_-induced apoptosis in INS-1 cells. Treatment with PCS extract inhibited cell death. PCS extract treatment decreased reactive oxygen species level and activated antioxidative enzymes. Among the major components of PCS extract, psoralen and isopsoralen (coumarins), but not bakuchiol, showed preventive effects against H_2_O_2_-induced beta-cell death. These findings indicate that PCS extract may be a potential pharmacological agent to protect against pancreatic beta-cell damage caused by oxidative stress associated with diabetes.

## 1. Introduction

It is commonly known that beta-cell death plays an important role in the pathophysiological progression of both type 1 and type 2 diabetes [1–3]. Although the main cause is not yet clear, glucolipotoxicity, cytokines, and genetic factors are associated with beta-cell apoptosis [4]. These stimuli are known to induce oxidative stress leading to apoptosis of the pancreatic beta-cells [5–7]. In this regard, treatment with antioxidant agents such as N-acetylcysteine, resveratrol, and *α*-lipoic acid showed cytoprotective effects on dysfunctional or apoptotic beta-cells [7–9]. In addition, oxidative stress is involved in the progression of diabetic complications including cardiomyopathy, nephropathy, neuropathy, retinopathy, and vascular damage [10].

The seeds of* Psoralea corylifolia *(PCS), commonly known as “Boh-Gol-Zhee” in Korea, have been used in traditional medicine. Six compounds, bakuchiol, psoralen, isopsoralen, corylifolin, corylin, and psoralidin, are the major components of PCS extract and are potent antioxidants [11]. PCS extract is used to treat diseases such as leucoderma [12] and impotence [13] and has antitumor [14] and antibacterial effects [15, 16]. In particular, bakuchiol, a polyphenol compound in PCS, has protective effects against hepatic injury [17, 18]. Similarly, psoralen and isopsoralen, coumarins of PCS, have antitumor effects [19] and alleviate amnesia [20]. However, PCS extract has not been used for the treatment of diabetes, and the effect of PCS on beta-cells is not known. In this study, we examined whether PCS extract has preventive effects against beta-cell damage in streptozotocin- (STZ-) induced diabetes in mice and beta-cell apoptosis* in vitro* to determine whether it might be a potential pharmacological treatment for diabetes.

## 2. Materials and Methods

### 2.1. Materials

RPMI1640 medium and fetal bovine serum were purchased from Gibco BRL (Grand Island, NY). Antibodies against catalase, heme oxygenase- (HO-)1, and phosphorylated 5′ AMP-activated protein kinase *α* (pAMPK*α*) were obtained from Santa Cruz Biotechnology Inc. (Santa Cruz, CA). Antibodies against cleaved poly (ADP-ribose) polymerase (PARP), pro-PARP, cleaved caspase3, procaspase3, phosphorylated c-Jun N-terminal kinase (pJNK), and phosphorylated-p38 were obtained from Cell Signaling (Boston, MA). An antibody against actin was obtained from Sigma-Aldrich (St. Louis, MO). Horseradish peroxidase-conjugated secondary antibodies were obtained from Santa Cruz Biotechnology Inc. Bakuchiol was purchased from Enzo Life Sciences Inc. (Farmingdale, NY). STZ, resveratrol, psoralen, and isopsoralen were obtained from Sigma-Aldrich (St Louis, MO).

### 2.2. Preparation of PCS Extract

The PCS used in the present study was purchased from an oriental drug store (Kwang Myung Dang Co., Ulsan, Korea), and the voucher specimen was deposited in the Herbarium of Korea Institute of Oriental Medicine (KIOM) under the registration number KIOM-111930. The extract was prepared by the standard procedure. In brief, the dried seeds (300 g) were ground into small pieces and then extracted twice with distilled water under reflux. The combined water extract was evaporated in vacuo to give a dark brownish residue (61.92 g).

### 2.3. Cell Culture

INS-1 cells were maintained at subconfluence at 37°C with 5% CO_2_. The cells were grown in RPMI1640 with 10% fetal bovine serum containing 50 *μ*M* beta*-mercaptoethanol and 100 units/mL of penicillin and streptomycin. Cells were incubated with PCS extract (6.25–400 *μ*g/mL) or purified individual components of PCS extract (0.5–4 *μ*g/mL) for 24 h. H_2_O_2_ (100 *μ*M) was added for the last 30 minutes or 1 hour of incubation. Resveratrol (50 *μ*M) was used as a positive control.

### 2.4. Animals

Six-week-old male C57BL/6 mice were supplied by the Orient Bio Inc. [13]. Animals were maintained at animal facilities at the Lee Gil Ya Cancer and Diabetes Institute, Gachon University of Medicine and Science, under a 12-h light and 12-h dark photoperiod. All animal experiments were carried out under a protocol approved by the Institutional Animal Care and Use Committee at Lee Gil Ya Cancer and Diabetes Institute, Gachon University. After adaptation for one week, mice were given an injection of 50 mg/kg/day i.p. STZ for 5 consecutive days. Age-matched control mice received an equal volume of vehicle. STZ-injected mice were treated orally with PCS extract (200 or 500 mg/kg/day) or vehicle at the same as the STZ injections began, and treatments continued daily for 8 weeks.

### 2.5. Glucose Tolerance Tests

Animals were fasted overnight and glucose (2 g/kg) was administered by intraperitoneal injection. Blood samples were obtained from the tail vein at 0, 30, 60, 90, 120, 150, and 180 min after glucose load. Blood glucose levels were measured with a glucose analyzer (OneTouch Ultra, Lifescan, Johnson & Johnson, Milpitas, CA).

### 2.6. Measurement of Hemoglobin A1c (HbA1c) Levels

HbA1c measurements were made using an AU 680 chemistry analyzer (Beckman Coulter, Inc., Brea, CA) and an HbA1c APT kit (Beckman Coulter, Inc.) following the manufacturer's instructions. HbA1c < 6% is considered normal [21].

### 2.7. Measurement of Blood Glucose and Serum Insulin

Blood samples were collected to measure blood glucose and serum insulin. The blood sampling line was filled with a solution of 4.5% EDTA to prevent blood clotting. Samples were kept on ice, and plasma was isolated and stored at −70°C until analysis. Glucose levels were measured with a glucose analyzer (OneTouch Ultra). Insulin levels were determined in duplicate using 5 *μ*L of serum and an Ultrasensitive Mouse Insulin kit (ALPCO, Windham, NH) according to the manufacturer's instructions.

### 2.8. Immunohistochemical and Histological Staining of Pancreatic Sections

Mice were killed and pancreatic specimens were removed, fixed in 10% formalin, and embedded in paraffin. For insulin staining, frozen sections were incubated for 16 hours with guinea pig polyclonal antibodies against insulin (Dako, Carpenteria, CA) diluted 1 : 100 in 0.1% PBS containing 0.3% TritonX-100 at 4°C. Horseradish peroxidase-conjugated secondary antibodies were incubated for 1 hour at room temperature and visualized using diaminobenzidine (Dako). Slides were counterstained with haematoxylin dehydrated sequentially in ethanol, cleared with xylenes, and mounted.

### 2.9. Western Blotting

Cells were solubilized with mammalian protein extraction buffer (GE Healthcare, Milwaukee, WI) containing a protease and phosphatase inhibitor cocktail (Sigma-Aldrich). Proteins (30–50 *μ*g) were resolved by 8 or 15% sodium dodecyl sulfate polyacrylamide gel electrophoresis, transferred onto membranes, and blocked with trisbuffered saline containing Tween 20 in 5% nonfat dry milk. The membranes were incubated with specific primary antibodies and visualized by incubating with horseradish peroxidase-conjugated secondary antibodies. Chemiluminescence was detected by LAS-4000 (Fuji Film, Tokyo, Japan) after adding Immobilon Western chemiluminescent HRP substrate (Millipore, St. Charles, MO). The images derived from western blotting were analyzed through ImageJ (National Institutes of Health, Bethesda, MD) software for Windows. Each densitometric value was normalized to *β*-actin.

### 2.10. Reactive Oxygen Species Detection

For quantification of intracellular reactive oxygen species (ROS) levels, cells were loaded with 10 *μ*M 2′,7′-dichlorodihydrofluorenscein diacetate (Molecular Probes, Eugene, OR) for 30 min at 37°C and 5% CO_2_ in phosphate-buffered saline (PBS). Cells were collected and washed twice with PBS and suspended in 500 *μ*L PBS. Mean fluorescence intensity was used as a measure of ROS as determined by flow cytometry FACSCalibur (BD Biosciences, San Jose, CA) using CellQuest Pro 5.2 with an excitation wavelength of 485 nm and an emission wavelength of 530 nm.

### 2.11. Superoxide Dismutase and Glutathione Peroxidase Activity Measurements

Superoxide dismutase (SOD) and glutathione peroxidase [22] activities were determined using a Superoxide Dismutase assay kit and Glutathione Peroxidase assay kit, respectively, following the manufacturer's instructions (Cayman Chemical, Ann Arbor, MI).

### 2.12. Measurement of Viable Cell Numbers

The number of viable cells was measured by a CCK-8 assay kit (Dojindo Laboratories, Kumamoto, Japan). Briefly, 10 *μ*L of CCK-8 dye and 100 *μ*L of cell culture medium were added to each well and incubated for 2 h at 37°C. Plates were then analyzed with a VERSAmax microplate reader (Molecular Devices Sunnyvale, CA). Measurements of formazan dye absorbance were carried out at 450 nm, with the reference wavelength at 620 nm. The number of viable cells after each treatment was normalized as a percentage of the number of viable cells in the control group.

### 2.13. Liquid Chromatography-Mass Spectrometry Analysis

Liquid chromatography-mass spectrometry analysis of PCS extract was performed on an Ultimate 3000 HPLC system combined with a Q Exactive Orbitrap mass spectrometer from Thermo Fisher Scientific (Bremen, Germany). A CSH C18 column (70 × 2.1 mm, 1.7 *μ*m) (Waters, Milford, MA) was employed for reverse phase separations. The mobile phases were 0.1% v/v formic acid in water and 0.1% v/v formic acid in acetonitrile. The elution gradient for the CSH C18 condition was programed as increasing percentage of B from 0% to 40% in 9 minutes, holding at 100% of B for 1 minute, and finally reequilibrating the column at 0% for 3 minutes. The electrospray ionization interface was operated in positive polarity mode. The spray voltage was 3.5 kV. The temperature of the ion transfer capillary was 350°C and sheath and auxiliary gases were 50 and 10 arbitrary units, respectively. The full scan range was 50 to 750 m/z with automatic gain control target and resolution settings as 1e^6^ and 140,000, respectively. The selective MS^2^ fragmentation of psoralen and isopsoralen was carried out by using higher collisional induced dissociation at 60 V.

### 2.14. Statistical Analyses

All data are expressed as mean ± standard error of at least three independent experiments. Data were analyzed using Analysis of Variance followed by* posthoc* analysis using the Tukey range test (SPSS 10.0 statistical software). *P* values less than 0.05 were considered statistically significant.

## 3. Results

### 3.1. Effect of PCS Extract on Prevention of STZ-Induced Diabetes

**Figure 1:**
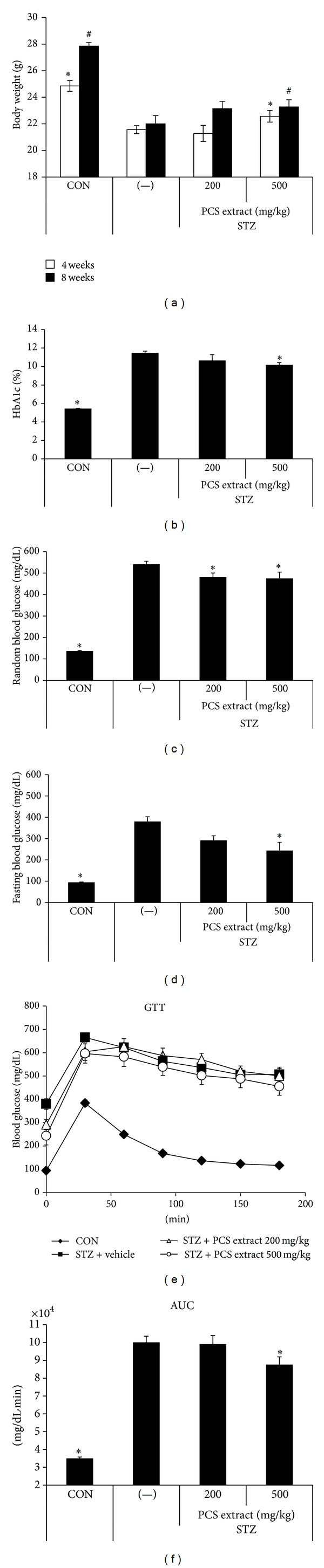
Amelioration of STZ-induced hyperglycemia by PCS extract. Mice were injected with vehicle (CON) or STZ (50 mg/kg/day) for 5 consecutive days. STZ-injected mice were treated with vehicle (−) or PCS extract (200 or 500 mg/kg/day) for 8 weeks beginning on the first day of STZ injection. (a) Body weight at 4 and 8 weeks after PCS extract treatment. *P < 0.05* versus* (−)/STZ mice at 4 weeks. ^#^ *P* < 0.05* versus* (−)/STZ mice at 8 weeks. (b) HbA1c levels. (c) Random blood glucose levels. (d) Fasting blood glucose levels after 8 weeks of PCS extract treatment. (e) Glucose tolerance test (GTT). Mice were fasted overnight and glucose (2 g/kg i.p.) was administered. Blood glucose levels were measured at the indicated times after glucose load. (f) Area under the curve (AUC) of the GTT graph. *P < 0.05* versus* (−)/STZ mice.

To determine the effect of PCS extract on the prevention of beta-cell damage, we injected STZ (50 mg/kg i.p. for 5 days) and administered PCS extract (200 or 500 mg/kg/day) for 8 weeks beginning on the same day as the first STZ injection and monitored body weight changes, HbA1c levels, and blood glucose levels. As shown in Figure 1(a), induction of diabetes by STZ caused a significant weight loss, and administration of PCS extract at 500 mg/kg/day significantly reduced the weight loss in STZ-injected mice at 4 and 8 weeks after treatment (Figure 1(a)). HbA1c levels were significantly lower in PCS extract-treated diabetic mice as compared with PBS-treated diabetic mice (Figure 1(b)). Similarly, both nonfasting (Figure 1(c)) and fasting (Figure 1(d)) blood glucose levels in PCS extract-treated diabetic mice were significantly lower than PBS-treated diabetic mice at 8 weeks after treatment.

To assess the effect on glucose regulation in PCS extract-treated diabetic mice, we performed intraperitoneal glucose tolerance tests. As shown in Figure 1(e), a glucose load given to the normal control group produced a rapid increase in blood glucose levels at 30 min which returned to baseline values within 120 min. After glucose loading, all STZ-induced diabetic mice showed hyperglycemia above 400 mg/dL at all measurement times. However, treatment of diabetic mice with 500 mg/kg/day of PCS extract resulted in a significant improvement in glucose tolerance compared with the vehicle-treated group (Figure 1(f)).

### 3.2. Effect of PCS Extract on Prevention of Pancreatic *β*-Cell Damage by STZ Treatment

**Figure 2:**
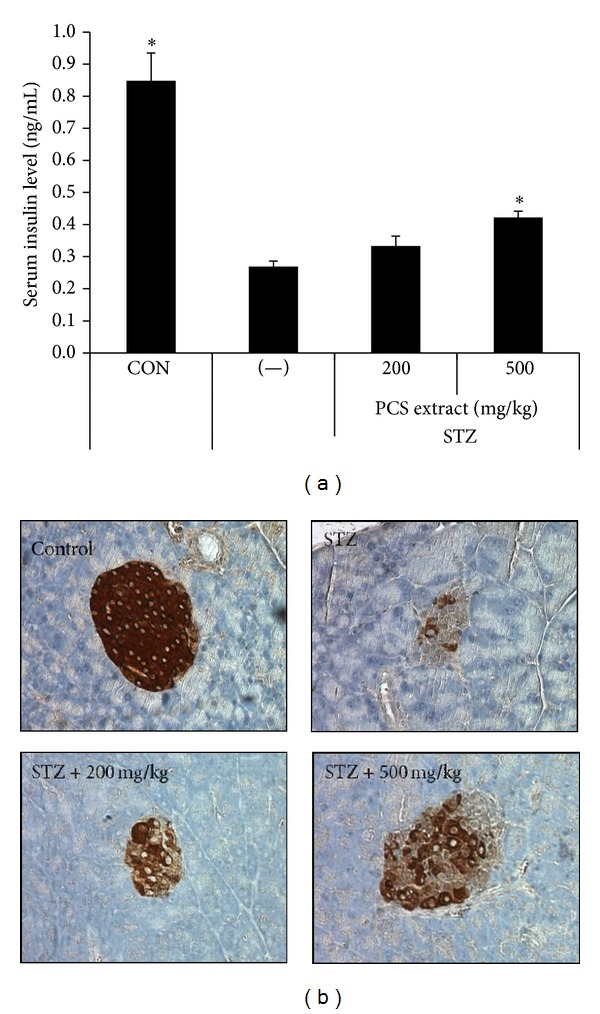
Reduction of STZ-induced beta-cell damage by PCS extract. Mice were injected with vehicle (CON) or STZ (50 mg/kg/day) for 5 consecutive days. STZ-injected mice were treated with vehicle (−) or PCS extract (200 or 500 mg/kg/day) for 8 weeks beginning on the first day of STZ injection. (a) Serum insulin level after 8 weeks of PCS extract treatment. *P < 0.05* versus* (−)/STZ mice. (b) Pancreatic sections stained with anti-insulin antibody.

As blood glucose levels were significantly lower in PCS extract-treated diabetic mice than in PBS-treated diabetic mice, we examined whether this effect was due to the reduction of pancreatic beta-cell damage caused by STZ injection. Serum insulin was significantly decreased in STZ-induced diabetic mice; however, administration of 500 mg/kg/day of PCS extract significantly inhibited this decrease of plasma insulin levels (Figure 2(a)). The pancreatic islets of vehicle-treated diabetic mice revealed small, atrophied islets that showed light staining with anti-insulin antibody, ambiguity of their verges, and degranulation of cells (Figure 2(b)). PCS extract-treated diabetic mice showed stronger staining with anti-insulin antibody, larger islets, and fewer degenerative changes compared with the PBS-treated group (Figure 2(b)).

### 3.3. Effect of PCS Extract on H_2_O_2_-Induced Apoptosis in INS-1 Cells

**Figure 3:**
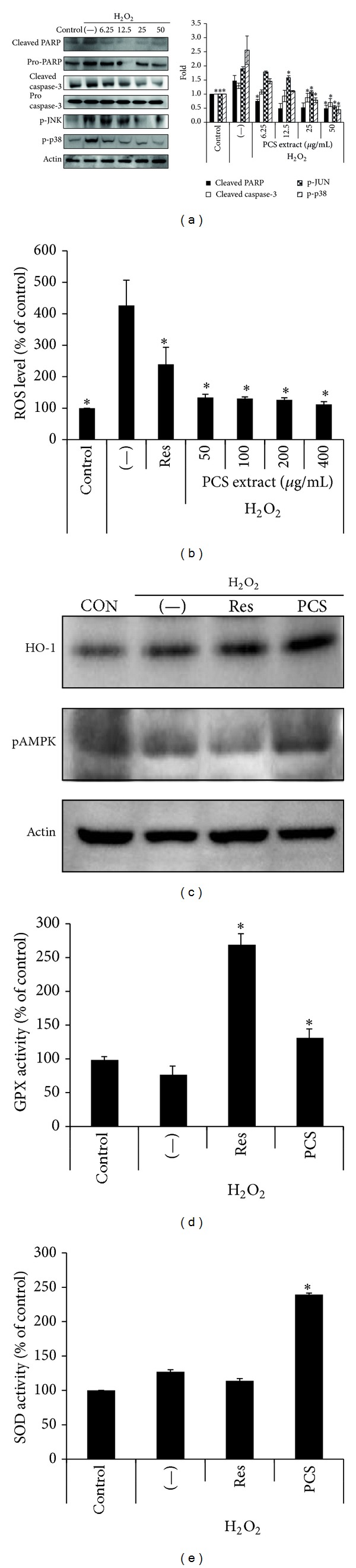
Antiapoptotic and antioxidative effect of PCS extract in H_2_O_2_-treated INS-1 cells. INS-1 cells were treated without (−) or with various doses of PCS extract for 24 h, and 100 *μ*M H_2_O_2_ was added for the last 1 h (a) or 30 min ((b)–(d)). (a) Protein levels of apoptotic signaling pathway were analyzed using western blotting. The representative blots (left) and the quantitative analysis of the band intensity by densitomery (right). The data are presented as a ratio of control. (b) ROS levels were measured. (c) Levels of anti-oxidative related proteins were analyzed using western blotting. (d), (e) GPX activity and SOD activity were measured. Resveratrol (Res) was used as a positive control. *P < 0.05* versus* (−)/H_2_O_2_.

To investigate whether PCS extract has protective effect on oxidative stress-induced beta-cell apoptosis, we examined the effects of PCS extract on cell death in H_2_O_2_-treated INS-1 cells. Western blot analysis of cleaved PARP and cleaved caspase-3, apoptosis-related proteins, showed that H_2_O_2_ treatment increased the expression of these proteins, and PCS extract pretreatment inhibited this increase in a dose-dependent manner. Mitogen-activated protein kinases (MAPK) are key molecules in the apoptotic signaling pathways, and ROS are known to induce MAPK signaling pathway activation [2]. Thus, we also checked phosphorylated p38 MAPK and p-JNK and found that the expression of these proteins was similarly inhibited by PCS extract pretreatment (Figure 3(a)).

### 3.4. Antioxidative Effects of PCS Extract in INS-1 Cells

To examine whether PCS extract has free radical scavenging effects, we measured the intracellular ROS level in INS-1 cells after treatment with H_2_O_2_ in the presence or absence of PCS extract. The ROS level was significantly reduced by 50 *μ*g PCS extract and was not further reduced by higher doses of PCS extract (Figure 3(b)). Antioxidative enzymes such as GPX and SOD remove ROS and protect cells from oxidative damage, and AMPK is known to maintain metabolic homeostasis. Heme oxygenase-1 (HO-1) is also an antioxidative protein induced in response to a variety of oxidative stresses. We found that HO-1 and pAMPK protein levels were increased by the addition of PCS extract (Figure 3(c)). The activities of GPX (Figure 3(d)) and SOD (Figure 3(e)) were also unchanged by H_2_O_2_ treatment alone but were significantly increased by the addition of PCS extract.

### 3.5. Effects of Bakuchiol, Psolaren, and Isopsolaren on H_2_O_2_-Induced Beta-Cell Death

**Figure 4:**
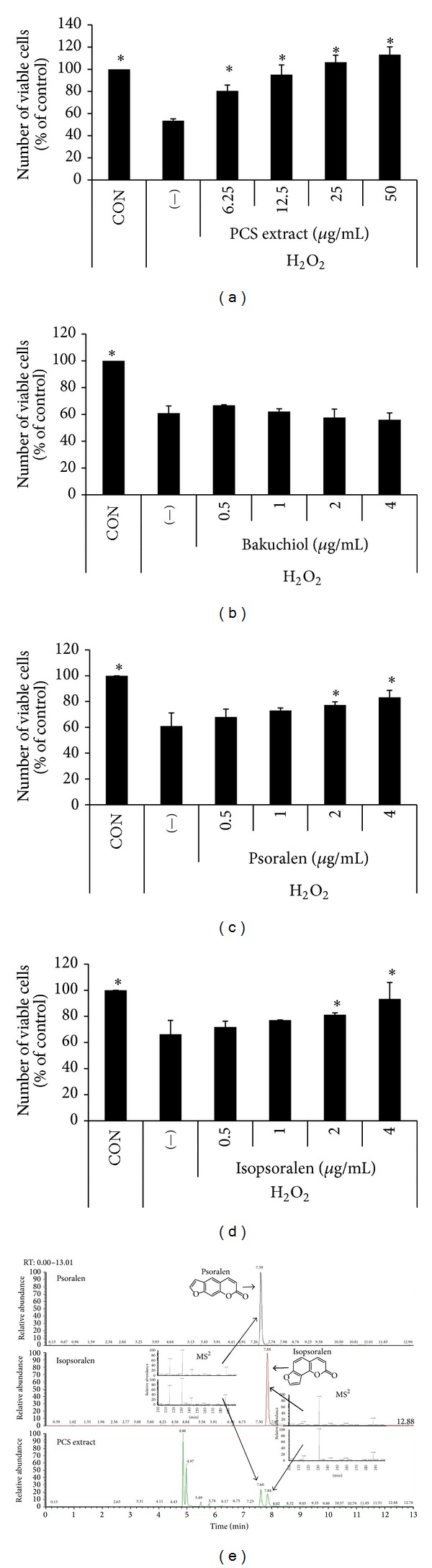
Antiapoptotic effect of psolaren and isopsolaren, PCS extract components, in H_2_O_2_-treated INS-1 cells. (a)–(d) INS-1 cells were treated without (−) or with various doses of PCS extract (a) or components of PCS extract ((b) bakuchiol, (c) psoralen, and (d) isopsoralen) for 24 h, and 100 *μ*M H_2_O_2_ was added for the last 1 h. Cell viability was measured by a CCK-8 assay kit. *P < 0.05* versus* (−)/H_2_O_2_. (e) Extracted ion chromatograms and MS^2^ spectrums for psoralen and isopsoralen. Ion chromatograms ([M + H] = 187.0386) were extracted from psoralen (1 *μ*g/mL), isopsoralen (1 *μ*g/mL), and PCS extract (100 *μ*g/mL) chromatograms. MS^2^ spectrums were obtained from each compound and PCS extract.

Bakuchiol, psoralen, and isopsoralen are known to be active components of PCS extract. To determine whether any of these compounds might be responsible for the antiapoptotic effect of PCS extract in H_2_O_2_-induced beta-cell death, we examined the antiapoptotic effects of purified bakuchiol, psoralen, and isopsoralen on INS-1 cells. As shown in Figure 4, INS-1 cells treated with H_2_O_2_ showed reduced cell viability by approximately 60% relative to control cells. When the cells were pretreated with PCS extract, the cell survival rate increased dose dependently (Figure 4(a)). The H_2_O_2_-induced cytotoxic effect was not affected by cotreatment with bakuchiol (Figure 4(b)) but was significantly attenuated by cotreatment with psoralen (Figure 4(c)) or isopsoralen (Figure 4(d)).

### 3.6. Identification of Psoralen and Isopsoralen in PCS Extracts

To determine whether psoralen and isopsoralen are present in the PCS extracts used in this study, PCS extracts (100 *μ*g/mL) were run on the HPLC-Q Exactive Orbitrap mass spectrometer in a scan and MS2 mode (Figure 4(e)). Extracted ion chromatogram ([M + H] = 187.0386) for psoralen and isopsoralen showed that both compounds were present in our PCS extracts. Psoralen and isopsoralen were separated and eluted at 7.59 min and 7.84 min, respectively. The MS^2^ spectrum for psoralen and isopsoralen further confirmed that both compounds were present in PCS extracts.

## 4. Discussion

*Psoralea corylifolia* L. seeds (PCS), commonly known as “Boh-Gol-Zhee” in Korea, have been used in traditional medicine for joint and back pain as well as other conditions. These seeds have been widely studied for their antitumor, antioxidant, antimicrobial, and anti-inflammatory effects [12, 15, 19, 23]. In our previous study, PCS extract also showed antisenescence effects in human dermal fibroblasts [24]. It is well known that oxidative stress is related to cellular senescence and aging [25]. Similarly, most components of PCS are potent antioxidants [11, 23, 26]. The antioxidant activities of the compounds decrease in the following order: psoralidin > bakuchiol > corylifolin > corylin > isopsoralen = psoralen [11]. The antioxidative properties of PCS make it of interest with regard to possible beneficial effects on oxidative stress-related diseases.

In both type 1 and type 2 diabetes, oxidative stress is thought to mediate beta-cell death, by inflammatory processes in the case of type 1 diabetes and by chronic hyperglycemia in the case of type 2 diabetes [5, 7, 27]. Diabetes increases the production of tissue-damaging ROS by glucose autoxidation and nonenzymatic protein glycosylation. Thus, protecting beta-cells against damage induced by oxidative stress is an important strategy for the treatment of diabetes. PCS have been used traditionally as a medicine in Asia and are known to have antioxidant activity [23, 26]. In this study, we investigated the protective effects of PCS extracts against beta-cells apoptosis induced by oxidative stress using STZ-induced diabetic mice as an animal model. Although this animal model does not mimic either the autoimmune destruction of beta-cells found in type 1 diabetes or the insulin resistance found in type 2 diabetes, STZ does substantially increase the ROS level in the pancreas and cause selective pancreatic beta-cell death [28, 29], thus providing a model in which the possible protective effect of PCS can be studied.

We found that STZ injection into C57BL/6 mice led to body weight loss, hyperglycemia, and hypoinsulinemia, all of which were significantly ameliorated by administration of PCS extract at 500 mg/kg/day. Although significant, the effects of PCS on blood glucose were relatively mild and did not completely restore normoglycemia. Furthermore, our histological study showed a protective effect of PCS extract on pancreatic islet structure and insulin staining. These data suggest that PCS extract reduces the diabetic symptoms and pancreatic damage induced by STZ.

Oxidative stress can activate mitogen-activated protein kinase (MAPK) cascades, including JNK and p38 MAPK [30], and antioxidant treatment prevents MAPK activation and beta-cell apoptosis [31]. These proteins mediate many important signals in mammalian cells, including cell proliferation, differentiation, and apoptosis. Similarly, MAPK activation contributes to pancreatic beta-cell dysfunction and apoptosis through PARP and capase-3 cascade signaling. We found that pretreatment of INS-1 cells with PCS extract inhibited the phosphorylation of JNK and p38 MAPK and also decreased the cleavage of PARP and caspase-3 proteins, which are major apoptotic signaling molecules [32]. This suggests that PCS extract has antiapoptotic effect in beta-cells.

To investigate the protective mechanisms of PCS extract, we used an H_2_O_2_-induced apoptosis cell model. Our results demonstrated that treatment of INS-1 cells with H_2_O_2_ significantly increased intracellular ROS levels, and PCS extract inhibited H_2_O_2_-induced ROS generation. Cells are protected from oxidative stress by endogenous antioxidative enzymes such as GPX, SOD, and HO-1. Overexpression of antioxidant enzymes in the beta-cells of transgenic mice is extremely effective in preventing STZ-induced beta-cell death [22]. We observed that treatment of INS-1 cells with PCS extract increased the activity of GPX and SOD and increased the expression of HO-1 protein. In addition, phosphorylation of AMPK which is a well-known metabolic regulator was also increased by PCS treatment. This suggests that PCS extract reduces ROS by increasing the production of antioxidant enzymes, thus reducing oxidative stress.

ROS are by-products of normal cellular oxidative stress process. Most ROS are generated in the mitochondria. Several studies have demonstrated that the ROS generation performs a crucial function in promoting proapoptotic activity [7]. Mitochondrial proapoptotic signaling is related to caspase-3 and PARP activation [32], which was reduced by PCS extract in our study. Our previous study showed that PCS extract has a mitochondrial protective effect in hepatocytes [24]. Therefore, it is thought that PCS extract may have a mitochondrial protective effect in pancreatic beta-cells. Further studies are required to discover the detailed mechanisms.

We found that PCS extract prevented H_2_O_2_-induced beta-cell apoptosis in INS-1 cells. Bakuchiol, psoralen, and isopsoralen are major components of PCS extract and are the most studied components. We found that pretreatment of INS-1 cells with purified psoralen or isopsoralen (which are both coumarins), but not bakuchiol, inhibited H_2_O_2_-induced cell death. According to our liquid chromatography-mass spectrometry data, the PCS extract used in this study contained both psoralen and isopsoralen, suggesting that these coumarins are among the active components of PCS extract with respect to beta-cell protection. In our previous study, bakuchiol showed protective effects on H_2_O_2_-induced oxidative stress in hepatocytes [24], whereas it was ineffective in beta-cells in the present study. This suggests that different mechanisms might be involved in the protective effects against oxidative stress-induced apoptosis in insulin producing beta-cells (coumarin-mediated) compared with other cell types such as hepatocytes (bakuchiol-mediated).

In conclusion, PCS extract showed protective effects against STZ-induced pancreatic beta-cell damage* in vivo* and H_2_O_2_-induced apoptosis* in vitro*. Our data suggest that PCS extract has beneficial effects in pancreatic beta-cell destruction through its antioxidant and antiapoptotic actions. PCS extract may be a beneficial plant-based dietary component to counteract oxidative stress-induced beta-cell damage.

## Abbreviations

GPX: Glutathione peroxidase
HO: Heme oxygenase
MAPK: Mitogen-activated protein kinase
pAMPK*α*: Phosphorylated 5′ AMP-activated protein kinase *α*
PARP: Poly(ADP-ribose) polymerase
PBS: Phosphate buffered saline
PCS: * Psoralea corylifolia* seed
pJNK: Phosphorylated c-Jun N-terminal kinase
ROS: Reactive oxygen species
SOD: Superoxide dismutase
STZ: Streptozotocin.
